# Anatomy-guided resections for paralimbic tumors in the temporo-insular region: combining tumor and epilepsy surgery concepts

**DOI:** 10.3389/fneur.2024.1450027

**Published:** 2024-10-15

**Authors:** Emad Alkassm, Alexander Grote, Björn Berger, Friedrich G. Woermann, Tunc Faik Ersoy, Roland Coras, Thilo Kalbhenn, Matthias Simon

**Affiliations:** ^1^Department of Neurosurgery, Evangelisches Klinikum Bethel, Universitätsklinikum Ostwestfalen-Lippe, Bielefeld, Germany; ^2^Department of Neuroradiology, Evangelisches Klinikum Bethel, Universitätsklinikum Ostwestfalen-Lippe, Bielefeld, Germany; ^3^Department of Epileptology, Krankenhaus Mara, Universitätsklinikum Ostwestfalen-Lippe, Bielefeld, Germany; ^4^Institute of Neuropathology, University of Erlangen, Erlangen, Germany

**Keywords:** paralimbic, temporo-mesial, insula, supramarginal resection, epilepsy surgery

## Abstract

**Object:**

Tumors in the temporo-mesial region often extend into the insula and vice versa. The present study investigated the results of a surgical strategy that combines principles of tumor and epilepsy surgery.

**Methods:**

We retrospectively analyzed 157 consecutive patients with intrinsic brain tumors in the temporo-mesial region, with varying degrees of extensions into the insula (44 patients, 28.0%). The surgical strategy utilized “anatomy-guided resection,” targeting specific anatomical compartments infiltrated by the tumor (e.g., temporal pole, anterior temporo-mesial region = uncus and hippocampal head, posterior temporo-mesial, insula) rather than treating the tumor as a single mass.

**Results:**

The most frequent histologies were ganglioglioma CNS WHO grade 1 (55 patients, 35.0%) and IDH1 wildtype glioblastoma (36 patients, 22.9%). Tumor infiltration was most commonly found in the anterior temporo-mesial compartment (145 patients, 92.4%). An anterior temporal lobectomy was part of the surgical strategy in 131 cases (83.4%). Seventy-six patients (48.4%) with drug-resistant epilepsy underwent a formal presurgical epilepsy work-up, including depth electrode placement in three cases. Complete resections were achieved in 117 patients (74.5%), with supramarginal resections performed in 89 cases (56.7%). Four patients experienced non-temporary neurological complications (CTCAE grade 3–5). At 6 months, 127 of 147 assessable patients (86.4%) were free from seizures or auras (ILAE class 1), excluding early postoperative seizures (<30 days). At 24 months, 122 of 144 assessable cases (84.7%) remained seizure-free (ILAE class 1). Kaplan–Meier estimates for 5-year overall survival were 98.5% for non-recurrent glioneuronal tumors. The 2-year overall survival estimates were 96.0% for 24 primary diffuse CNS WHO grade 2 and 3 gliomas and 55.2% for 30 patients undergoing first surgeries for glioblastomas/astrocytomas CNS WHO grade 4.

**Conclusion:**

Combining both epilepsy and tumor surgery concepts in the surgical treatment of intrinsic brain tumors involving the mesial temporal lobe, often extending into the insula, led to more extensive resections, improved seizure outcomes, and potentially even better patient survival outcomes.

## 1 Introduction

Temporo-mesial gliomas often involve the insula (and vice versa) rather than growing into the lateral aspects of the temporal lobe. This growth pattern is believed to reflect the phylogeny of the brain. Yasargil coined the term “paralimbic gliomas” to describe such growths (and other neoplasms) affecting the phylogenetically older parts of the brain located between and connecting the limbic system with the phylogenetically younger neocortical areas (1). Temporo-mesial gliomas, with and without the involvement of the insula, might thus be considered antero-inferior paralimbic gliomas.

The specific growth patterns and extensions of these tumors significantly impact surgical strategies. It is widely accepted that addressing tumorous infiltration in various parts of the temporal lobe, the insula, and other paralimbic regions (such as the fronto-orbital area) requires different surgical approaches and concepts, which must be combined into a unified strategy for each patient. Classification schemes can aid in this decision-making process. Both temporal lobe and insular gliomas have garnered considerable neurosurgical attention. Paralimbic gliomas of the temporal lobe are sometimes referred to as temporal mediobasal tumors (1).

The classification of temporal mediobasal tumors proposed by Aliashkevich et al. (2) recognizes a possible insular extension of the growths. Yasargil’s classification of insular gliomas is based on the concept of the paralimbic system mentioned above and, therefore, describes in some detail tumor growth beyond the boundaries of the insula, among other things, into the temporal operculum and temporo-mesiobasal structures (1, 3). Pitskhelauri and co-workers also recognize temporal lobe involvement by insular tumors (4). In contrast, the classification scheme for insular gliomas proposed by Sanai et al. (5) describes the infiltration of parts of the insula solely and does not mention extrainsular tumor growth.

Surgical treatment for tumors of the temporal region and their insular extensions can be challenging since these lesions often border on language, motor, and visual pathway eloquent cortices and white matter tract (2–6). Therefore, surgery for these growths may carry considerable neurological risks. With respect to neurological complications, intraoperative manipulation and management of blood vessels is often the most prominent concern (3).

The clinical course of patients with tumors in the temporo-insular region is frequently complicated by epilepsy. Sometimes, epilepsy control is the dominant clinical concern, and these cases are often referred to epilepsy surgery centers. The epilepsy surgery perspective differs from the tumor surgery view. Epilepsy surgery usually aims at removing what is called the epileptogenic zone, i.e., the part of the brain responsible for seizure generation. In tumor cases, the epileptogenic zone usually includes the neoplasm and a variable amount of non-tumorous (cortical) tissues surrounding the growth (7). For example, patients with pharmacoresistant epilepsy and an anterior temporal lobe tumor will often undergo an anterior temporal lobectomy (ATL) for epilepsy control. In oncological terms, this surgery would correspond to a supramarginal resection (8, 9). Supramarginal tumor surgery is currently receiving widespread attention and has been associated with superior oncological outcomes in both malignant and low-grade gliomas (10–12).

For some time, we have treated tumors of the lower anterior part of the paralimbic system (i.e., mesial temporal lobe with and without infiltration of the insular region), aiming at both epilepsy and tumor control. We employed a surgical strategy that addresses various anatomical compartments (“anatomy guided resection”) defined by specific surgical maneuvers necessary for resecting the respective tumor infiltration and/or presumably epileptogenic tissues. We reviewed our pertinent experience for the present study to assess resection and epileptological outcomes, surgical risks and complications.

## 2 Patients and methods

### 2.1 Patients and clinical data

For this analysis, we have searched our institutional neuro-oncology (2016–2021) and epilepsy surgery database (2013–2021) for all cases undergoing surgery in our department for an intrinsic brain tumor involving the anterior lower paralimbic structures of the temporal lobe with variable involvement of the insula. We identified 157 cases operated by three surgeons specializing in epilepsy and tumor surgery (TK: 2013–2022, AG: 2017–2022, MS: 2016–2022). We performed the respective surgeries adhering to the anatomy-guided resection principles outlined below.

Pertinent clinical data, including follow-up information, were collected retrospectively from the patients’ charts and through telephone interviews. We assessed functional outcomes using the postsurgical (discharge) Karnofsky Performance Index (KPI) and Neurologic Assessment in Neuro-Oncolog (NANO) scores, as well as the occurrence of surgical, neurological, or medical complications. The Common Terminology Criteria for Adverse Events (CTCAE) scheme was employed to grade the severity of complications. Neurological complications were recorded as temporary if they resolved within 30 days (13). Preoperative seizure history and any postoperative seizures were recorded together with the respective time-point. For this study, we analyzed EPS (early postoperative seizures; <30 days) (14), as well as epilepsy outcomes at 6 and 24 months following surgery using the International League Against Epilepsy (ILAE) and Engel epilepsy surgery outcome classifications (15). Briefly, ILAE class 1 refers to complete absences of seizures or auras, while Engel class I patients are free of “disabling seizures.” Drug-resistant epilepsy was defined according to the 2009 ILAE criteria.

The study was approved by the responsible institutional review board for human research and ethics committee (Ethikkommission der Ärztekammer Westfalen-Lippe und der Westfälischen Wilhelms-Universität Münster, Germany, Az 2018-143-f-S and Az 2020–368-f-S).

### 2.2 Surgical and radiological data

**Figure 1:**
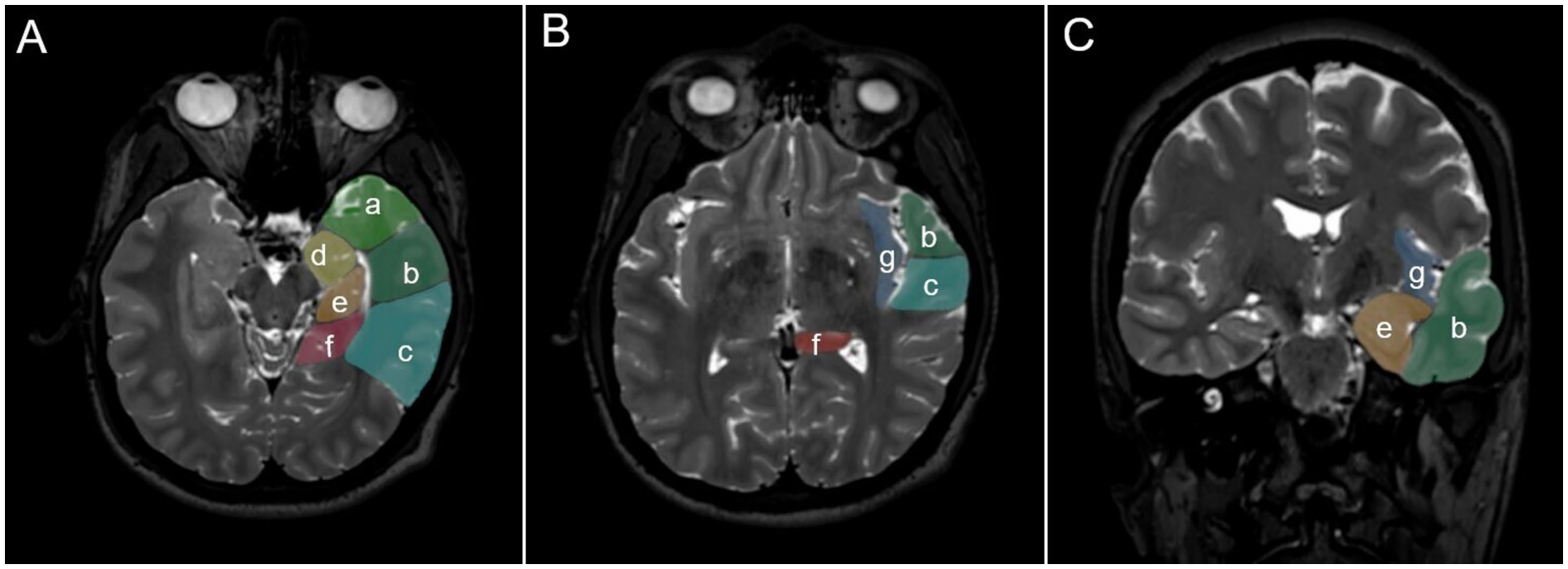
Illustration showing various anatomical compartments of the temporo-insular region, which are defined by specific surgical maneuvers necessary for their removal (“anatomy guided resection”). **(A,B)** Axial, **(C)**, coronal view: (a) temporal pole; (b) anterior (corresponding to the neocortical resection volume removed for an anterior temporal lobectomy) vs. (c) posterior lateral temporal lobe (with its dorsal border defined superiorly by the temporal language areas as assessed by intraoperative mapping or functional MRI studies and basally by the preoccipital notch); (d) anterior temporo-mesial region (uncus, amygdala, hippocampal head and parahippocampal gyrus up to the level of the choroidal point); (e) posterior temporo-mesial region (hippocampal body and parahippocampal gyrus posteriorly to the choroidal point and up to the level of the isthmus); (f) isthmus and posterior cingulum (up to the level of the corpus callosum); (g) insula.

All preoperative magnetic resonance imaging (MRI) investigations were reviewed (using contrast enhanced T1 weighted scans in cases with glioblastomas and fluid attenuated inversion recovery [FLAIR]/T2 sequences in all other patients) in order to assess tumor infiltration of the following compartments (Figure 1): temporal pole, anterior temporo-mesial region (uncus, amygdala, hippocampal head and parahippocampal gyrus up to the level of the choroidal point), posterior temporo-mesial region (hippocampal body and parahippocampal gyrus posteriorly to the choroidal point and up to the level of the isthmus), isthmus and posterior cingulum (up to the level of the corpus callosum), and anterior (corresponding to the neocortical resection volume removed for an anterior temporal lobectomy) vs. posterior lateral temporal lobe (with its dorsal border defined superiorly by the temporal language areas as assessed by intraoperative mapping or functional MRI studies and basally by the preoccipital notch) (16). The definition of these compartments reflects in part specific epilepsy surgery maneuvers that allow the performing of anatomical resections and thereby remove and address tumor infiltration of the various parts of the temporal lobe (7). We considered all temporal lobe tissues medial to a vertical imaginary plane from the insular cistern to the lateral boundary of the ventricle down to the skull base as mesial, and all tissues lateral to this plane as lateral since the authors perform a selective temporomesial resection using the lateral aspect of the temporal horn for guidance. We also assessed infiltration of the insula and other paralimbic areas, such as the orbito-frontal lobe. At least in principle, anatomy-guided resection of the insula and the frontal lobe is part of the (epilepsy) neurosurgery armamentarium.

The planned resection strategy varied with the individual tumor growth pattern and seizure history but not with the presumptive tumor histology *per se*. Surgeries are generally aimed at the removal of all tumor-infiltrated compartments as well as the presumed epileptogenic zone (if safely possible) (7). Postoperative imaging data were similarly reviewed in order to assess the overall degree of resection (including computer-assisted volumetric analyses [iplanNet, Brainlab AG, Munich, Germany]; complete: 0% residual tumor vs. incomplete). We specifically noted if the resection involved presumably non-tumorous tissues/anatomical compartments without tumor infiltration surrounding the growth for epilepsy (“epilepsy surgery”) and/or tumor control purposes (termed “supramarginal resections” if postoperative imaging showed no residual tumor).

### 2.3 Statistical analysis

We utilized routine statistical analyses and appropriate software (IBM SPSS Statistics for Windows, Version 25.0, IBM Corp., Armonk, NY), and *p*-values of <0.05 were considered significant. Survival studies were performed using Kaplan–Meier estimates, and survival differences were tested for significance with the log-rank test.

## 3 Results

### 3.1 Patient demographics and histologies

The two most frequent histologies in our series were ganglioglioma CNS WHO grade 1 (55 [35.0%]) and (IDH 1 wildtype) glioblastoma (36 [22.9%]). 24 (15.3%) patients had surgery for recurrent disease. A detailed account is presented in Table 1.

**Table 1:** Histologies and demographics.

Histology	*N*	Sex (M/F)	Age (median, IQF)
IDH wildtype glioblastoma CNS WHO grade 4^*^	36 (22.9%)	24 (66.7%)/12 (33.3%)	71.5, 58.8–76.0
IDH mutant astrocytoma (“secondary glioblastoma”) CNS WHO grade 4^†^	2 (1.3%)	1/1 (50.0/50.0%)	55.0, NA
Anaplastic astrocytoma CNS WHO grade 3	12 (7.6%)	9 (75.0%)/3 (25.0%)	42.5, 31.3–55.5
(Diffuse) astrocytoma CNS WHO grade 2	12 (7.6%)	8 (66.7%)/4 (33.3%)	35.0, 30.0–47.3
(Anaplastic) oligodendroglioma CNS WHO grades 2/3^‡^	12 (7.6%)	5 (41.7%)/7 (58.3%)	46.0, 37.5–63.8
Pilocytic astrocytoma CNS WHO grade 1	4 (2.5%)	3 (75.0%/25.0%)	18.5, 11.3–19.0
Isomorphic astrocytoma CNS WHO grade 1	2 (1.3%)	2 (100%)/0	37.5, NA
Angiocentric glioma CNS WHO grade 1	1 (0.6%)	0/1 (0/100%)	24.0, NA
Gangliocytoma and ganglioglioma CNS WHO grade 1^§^	56 (35.7%)	30 (53.6%)/26 (46.4%)	14.0, 7.0–26.8
Atypical and anaplastic ganglioglioma CNS WHO grades 2/3^¶^	2 (1.3%)	2 (100%)/0	11.0, NA
Dysembryoplastic neuroepithelial tumor (DNT) CNS WHO grade 1	15 (9.6%)	13 (86.7%)/2 (13.3%)	26.0, 16.0–46.0
Glioneuronal or -vascular hamartoma	3 (1.9%)	1 (33.3%)/2 (66.7%)	39.0, NA
All	157 (100%)	98 (62.4%)/59 (37.6%)	35.0, 16.5–58.5

* incl. 1 “gliosarcoma”.

† incl. 1 “giant cell glioblastoma”.

‡ CNS grade 2: 1 and grade 3: 1.

§ gangliocytoma: 1, ganglioglioma: 55.

¶ CNS grade 2: 1 and grade 3: 1.

### 3.2 Tumor growth patterns

A total of 81 (51.6%) tumors were located in the left, with 76 (48.4%) located in the right hemisphere, and multifocal growth was observed in three cases (1.9%). The vast majority of cases were found to have tumor infiltration of the anterior temporo-mesial compartment (145 [92.4%]) and/or the temporal pole (102 [65.0%]; both 94 [59.9%]). Insular tumor was seen in 44 (28.0%) cases.

The involvement of the various anatomical compartments varied with tumor histology (Table 2). Temporal pole (60/74 [81.1%] vs. 42/83 [50.6%]; *p* < 0.001), anterior and posterior temporo-lateral (40/74 [54.1%] vs. 13/83 [15.7%]; p < 0.001 and 14/74 [18.9%] vs. 3/83 [3.6%]; *p* = 0.003), and insular infiltration (41/74 [55.4%] vs. 3/83 [3.6%]; *p* < 0.001) were more frequent in diffuse gliomas and glioblastomas vs. all other pathologies, while the cases from the latter category invariably showed involvement of the anterior temporo-mesial region (83/83 [100%] vs. 62/74 [83.8%]; *p* < 0.001).

**Table 2:** Histology, tumor localization, and spread.

Histology*		GBM	Gliomas CNS WHO grades 2–3	Other gliomas	Glio-neuronal tumors	Developmental lesions	All
Compartment		*N* = 38	*N* = 36	*N* = 7	*N* = 73	*N* = 3	*N* = 157
Temporal pole		30/38 (78.9%)	30/36 (83.3%)	4/7 (57.1%)	38/73 (52.1%)	0	102/157 (65.0%)
Temporo-lat.	Anterior	26/38 (68.4%)	14/36 (38.9%)	7/7 (100%)	10/73 (13.7%)	0	53/157 (33.8%)
	Posterior	8/38 (21.1%)	6/36 (16.7%)	4/7 (57.1%)	2/73 (2.7%)	0	17/157 (10.8%)
Temporo-mesial	Anterior	33/38 (86.8%)	29/36 (80.6%)	3/7 (42.9%)	73/73 (100%)	3/3 (100%)	145/157 (92.4%)
	Posterior	25/38 (65.8%)	12/36 (33.3%)	1/7 (14.3%)	32/73 (43.8%)	1/3 (33.3%)	74/157 (47.1%)
Other (para-limbic)	Isthmus/post. Cingulum	8/38 (21.1%)	3/36 (8.3%)	1/7 (14.3%)	4/73 (5.5%)	0	16/157 (10.2%)
	Insula	17/38 (44.7%)	24/36 (66.7%)	0	3/73 (4.1%)	0	44/157 (28.0%)
	Frontal lobe	3/38 (7.9%)	16/36 (44.4%)	0	1/73 (1.4%)	0	20/157 (12.7%)
Other	Basal ganglia/diencephalon and midbrain	9/38 (23.7%)	9/36 (25.0%)	1/7 (14.3%)	4/73 (5.5%)	0	23/157 (14.6%)
	Mulifocal/−centric	3/38 (7.9%)	0	0	0	0	3/157 (1.9%)

*Glioblastoma: IDH wildtype glioblastoma CNS WHO grade 4, IDH mutant astrocytoma CNS WHO grade 4; gliomas CNS WHO grades 2–3: anaplastic astrocytoma CNS WHO grade 3, diffuse astrocytoma CNS WHO grade 2, (anaplastic) oligodendroglioma CNS WHO grades 2/3; other gliomas: pilocytic astrocytoma CNS WHO grade 1, isomorphic astrocytoma CNS WHO grade 1, angiocentric glioma CNS WHO grade 1; glioneuronal tumors: gangliocytoma and ganglioglioma CNS WHO grade 1, atypical and anaplastic ganglioglioma CNS WHO grades 2/3, dysembryoplastic neuroepithelial tumor (DNT) CNS WHO grade 1; developmental lesions: glioneuronal or -vascular hamartoma.

### 3.3 Surgical management

**Figure 2:**
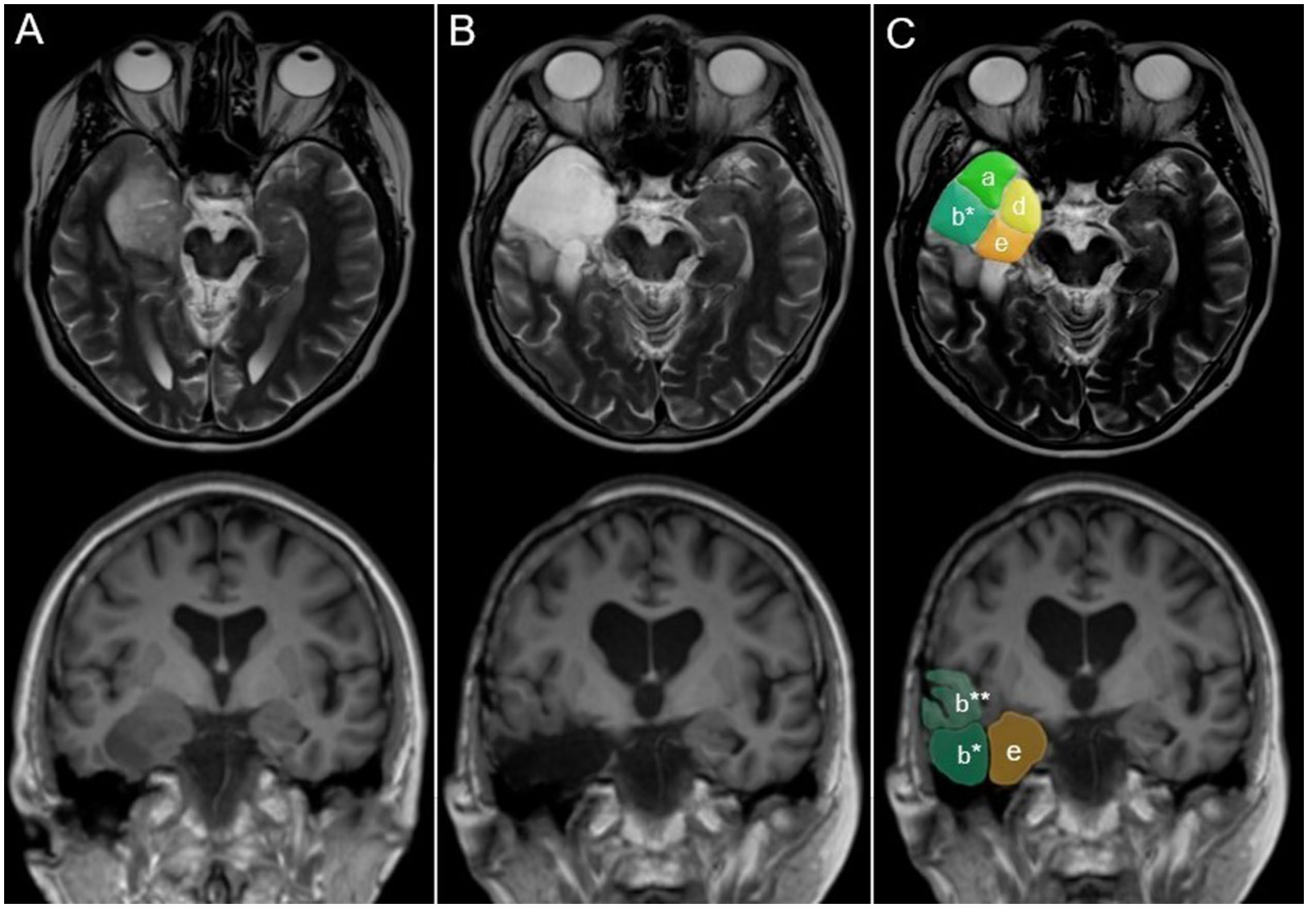
Anatomy-guided surgery for an anaplastic oligodendroglioma CNS WHO grade 3 in a 65-year-old patient. **(A)** Preoperative; **(B,C)** postoperative axial and coronal MR images. The tumor primarily involved (d) the anterior and (e) posterior temporo-mesial compartments and (a) the temporal pole. Aiming at both tumor and epilepsy control, the patient had a modified anterior temporal lobectomy, i.e., resection of the inferior part of the anterior temporal lobe (b*; superior part: b**) in addition to the compartments obviously infiltrated by the tumor (a,e,d). Compartment designations are the same as in Figure 1.

**Figure 3:**
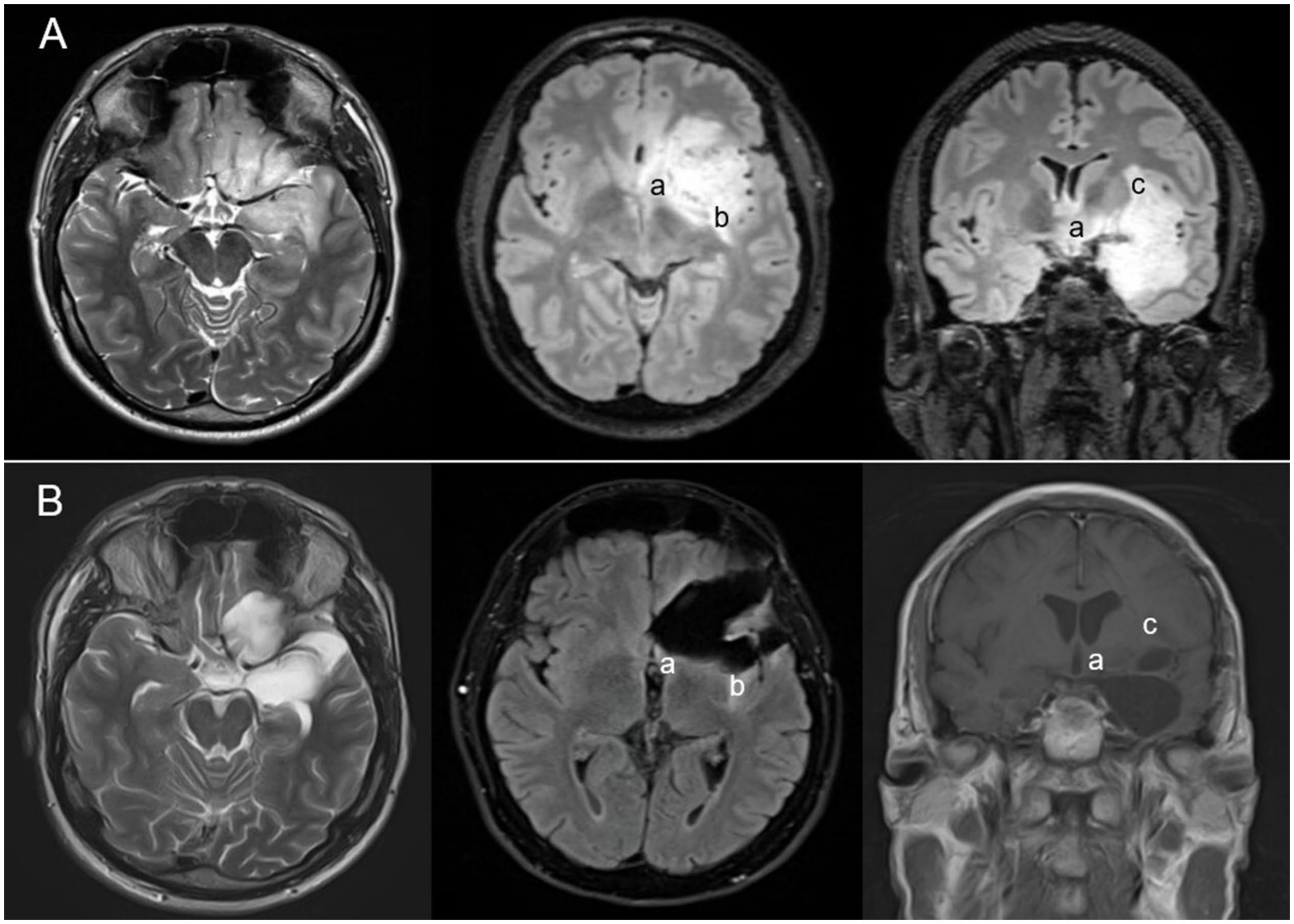
Anaplastic oligodendroglioma CNS WHO grade 3 in a 39-year-old patient with growth into paralimbic areas beyond the temporomesial region (e.g., insula and fronto-orbital cortices). Preoperative **(A)** and postoperative **(B)** MR scans. The tumor was operated combining epilepsy (modified ATL [anterior temporal lobectomy] = removal of the temporal pole and the anterior temporo-mesial compartment) and tumor surgery principles (maximal safe = transfrontal and transsylvian resection of tumor-infiltrated frontobasal and insular tissues; a residual tumor in projection on the anterior perforated substance [a], and in the dorsal [b] and superior insula [c]).

In 131 cases (83.4%), an anterior temporal lobectomy (ATL) was at least part of the surgical concept (Figure 2). Seventy-six patients (48.4%) had a standardized ATL, i.e., removal of 3.5–4 cm (left/dominant hemisphere) or ≈5 cm (right/non-dominant hemisphere) lateral temporal lobe tissues posterior to the temporal pole and resection of the mesial temporal pole, uncus, amygdalum, hippocampal head and a variable amount of the hippocampal body. A total of 55 (35.0%) cases had additional temporo-lateral and/or isthmic tissue removal and/or transfrontal and/or transsylvian insular surgery. In 14 patients (8.9%), the tumor was resected primarily through a transsylvian approach with the addition of separate frontal or temporal lobe corticotomies as required. Eleven cases (7.0%) were operated on using individualized transtemporal routes, including one patient who had surgery through a subtemporal route. In the remaining patient, posterior temporo-mesial and isthmic disease was addressed using a posterior paramedian parieto-occipital route. Insular tumor infiltration was addressed after a temporal lobe resection following the tumor through the temporal stem into the insula (3, 17), through the Sylvian fissure (11), or using a combination of these approaches (1) (Figure 3).

Intraoperative monitoring (continuous motor evoked potentials [MEP] recordings, 30–50% amplitude reduction threshold) and cortical/subcortical electrostimulation were employed in 62 cases (39.5%), including all insular surgeries. We feel that MEP monitoring helps to avoid (motor) deficits resulting from small vessel compromise, which is a prominent concern in insular surgery, more than temporo-mesial surgery (2, 3). We routinely respond to a relevant MEP amplitude reduction by relaxing any brain retractors, stopping the surgery, or continuing with a presumably safer part of the operation until the MEP recordings have returned to baseline. If these maneuvers do not suffice, we ask the anesthetist to elevate the patient’s mean arterial blood pressure (MAD) to >90–100 mmHg. In these latter cases (and in all patients with an early postoperative motor or language deficit), an elevated MAD is maintained for 72 h. following the surgery. We also feel that the risk of small vessel ischemia during insular surgery heavily depends on anatomy, i.e., where the surgery is performed within the insula. Insular resections are stopped when the level of the perforators originating from the proximal, middle cerebral artery (M1 segment) is reached, and the tumor close to the superior limiting sulcus is addressed very carefully, if at allon is avoided whenever possible when operating in the insula or close to the temporo-mesial arachnoid membranes.

Two patients had awake surgery and language mapping. We did not employ electrocorticography to help delineate the epileptogenic zone. Neuronavigation was utilized in all cases, and 5-aminolevulinic acid was administered per routine with presumed glioblastoma or CNS WHO grade 4 glioma. No additional intraoperative imaging techniques, such as ultrasound or MRI, were used to optimize the degree of resection.

**Figure 4:**
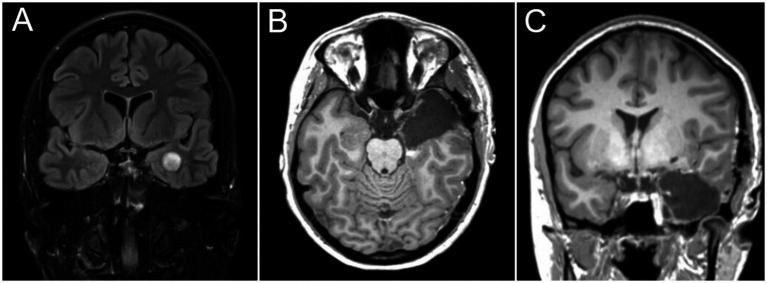
Surgical treatment (standard dominant-side anterior temporal lobectomy) for a ganglioglioma CNS WHO° I in a 22-year-old patient presenting with drug-resistant epilepsy. **(A)** Preoperative; **(B,C)** postoperative MRI scans.

A total of 126 (80.3%) were treated for “resectable,” i.e., unifocal-unilateral tumors, which did not infiltrate the basal ganglia, diencephalon, midbrain, or the primary motor or speech cortices/fiber tracts as assessed during awake surgery, through intraoperative electrophysiological mapping studies or functional MRI (fMRI). One hundred and seventeen cases (92.9% [74.5%] of the overall cohort) had a complete resection, including 21/23 (91.3%) “resectable” glioblastomas, 21/25 CNS WHO (84.0%) grade 2/3 gliomas, and 66/69 (95.7%) glioneuronal tumors (Figure 4). Insular tumor was completely resected in 19/44 cases (43.2, 50.0% of insular resections). Excluding cases with insular tumor extensions, 96/99 (97.0%) patients with “resectable” lesions had complete resections. Thirty-five patients (63.6%) of all non-recurrent cases with diffuse gliomas had a complete resection. We performed supramarginal resections in 89 patients (56.7% of all cases).

Resection outcomes varied depending on the anatomical compartment addressed (Table 3). The highest rates of complete or compartment resections were observed in the temporal pole and the anterior lateral and mesial temporal compartments (Figures 2, 4). Residual tumors were most commonly found in the basal ganglia, diencephalon, and midbrain (23/40 [57.5%]) and/or the insula (17/40 [42.5%]; Figure 3). In 104 cases (66.2%), we resected one, two, or three compartments (42, 46, and 16 cases, respectively) that were not involved by the tumor, including 84 anterior temporo-lateral compartments and 43 temporal poles, but no resections were performed in the frontal or insular compartments. All patients with drug-resistant epilepsy (DRE) underwent resections of compartments not infiltrated by the tumor, compared to 28 out of 81 patients (35.6%) in the remaining cohort. Specifically, 71 out of 73 patients (97.3%) with glioneuronal tumors had non-infiltrated compartments resected, compared to 13 out of 38 patients (34.2%) with glioblastomas or astrocytomas (CNS WHO grade 4).

**Table 3:** Tumor localization and resection outcomes.

Compartment		Not addressed	Residual tumor	Complete tumor resection	Compartment resection
Temporal pole		0	0	2/102 (2.0%)	100/102 (98.0%)
Temporo-lat.	Anterior	0	0	4/53 (7.5%)	49/53 (92.5%)
	Posterior	0	3/17 (17.6%)	11/17 (64.7%)	3/17 (17.6%)
Temporo-mesial	Anterior	0	0	7/145 (4.8%)	138/145 (95.2%)
	Posterior	0	2/74 (2.7%)	3/74 (4.1%)	69/74 (93.2%)
Other (para)limbic	Isthmus/post. Cingulum	3/16 (18.8%)	0	13/16 (81.3%)	0
	Insula	6/44 (13.6%)	19/44 (43.2%)	19/44 (43.2%)	0
	Frontal lobe	3/20 (15.0%)	2/20 (10.0%)	15/20 (75.0%)	0
Other	Basal ganglia/diencephalon and midbrain	23/23 (100%)	0	0	NA
	Multifocal/−centric	3/3 (100%)	0	0	NA

NA, not applicable.

### 3.4 Neurological and functional outcomes

Surgical mortality was 2/157 (1.3%, postoperative hemorrhage, 1, medical complications, 1). Four (2.6%) of the remaining 155 patients incurred non-temporary (i.e., persisting >30 days) neurological CTCAE grade 3–5 complications (hemiplegia). All four cases had insular resections (including two surgeries for recurrent disease) for diffuse gliomas CNS WHO grade 2 or 3. In three patients, the deficits were due to small perforating vessel compromise, as shown by postoperative MR imaging, and one had a postoperative bleed. Overall, 11 patients (7.1%) incurred CTCAE grade 3–5 surgical (postoperative hemorrhages requiring surgery: 4, surgeries for CSF fistula: 2, meningitis, all culture-negative: 5) and four (2.5%) CTCAE grade 3–5 medical complications. The median postoperative NANO score was 0 (IQR: 0–2; *cf.* preoperative NANO score: 0, IQR: 0–1), and the median postoperative KPI was 90 (IQR: 90–100; *cf.* preoperative KPI: 90, IQR: 90–100).

### 3.5 Epilepsy and epilepsy surgery aspects; epileptological outcomes

Seventy-six (48.4%) patients presented with drug-resistant epilepsy (DRE), while 34 patients (21.7%) reported no preoperative seizures. Seizure history varied with tumor histology and tumor infiltration of the various anatomical compartments defined above (Tables 4, 5). From an epilepsy surgery perspective, the operative concept addressed non-tumorous but presumably epileptogenic tissues in addition to the tumor in 106/123 (86.2%) cases presenting with seizures, including all 76 patients presenting with DRE. All DRE cases underwent a dedicated presurgical epileptological work-up, including video electroencephalogram (EEG) monitoring and specialized epilepsy neuroimaging. Three cases involved surgery for placement of depth electrodes that preceded the actual resective surgery.

**Table 4:** Tumor localization, preoperative epilepsy history, and epilepsy outcomes.

Compartment		Preoperative epilepsy			Epilepsy outcome	
		No seizures	Seizures, but not DRE	DRE	ILAE class 1 at 6 months*	ILAE class 1 at 24 months*
Temporal pole		29/102 (28.4%)	35/102 (34.3%)	38/102 (37.3%)	84/94 (89.4%)	79/92 (85.9%)
Temporo-lateral	Anterior	22/53 (41.5%)	19/53 (35.8%)	12/53 (22.6%)	42/45 (93.3%)	37/42 (88.1%)
	Posterior	3/17 (17.6%)	11/17 (64.7%)	3/17 (17.6%)	13/15 (86.7%)	12/14 (85.7%)
Temporo-mesial	Anterior	29/145 (20.0%)	40/145 (27.6%)	76/145 (52.4%)	118/137 (86.1%)	106/134 (79.1%)
	Posterior	16/74 (21.6%)	24/74 (32.4%)	34/74 (45.9%)	60/70 (85.7%)	54/68 (79.4%)
Other (para)limbic	Isthmus/cingulum	5/16 (31.3%)	6/16 (37.5%)	5/16 (31.3%)	14/15 (93.3%)	12/14 (85.7%)
	Insula	12/44 (27.2%)	29/44 (65.9%)	3/44 (6.8%)	34/36 (94.4%)	34/35 (97.1%)
	Frontal lobe	4/20 (20.0%)	15/20 (75.0%)	1/20 (5.0%)	16/18 (88.9%)	17/18 (94.4%)
Other	Basal ganglia/diencephalon and midbrain	3/23 (13.0%)	16/23 (69.6%)	4/23 (17.4%)	15/19 (78.9%)	14/18 (77.8%)
	Mulifocal/−centric	3/3 (100%)	0	0	2/2 (100%)	2/2 (100%)
All		34/157 (21.7%)	47/157 (29.9%)	76/157 (48.4%)	127/147 (86.4%)	122/144 (84.7%)

*Excluding early postoperative seizures (EPS).

**Table 5:** Histology, preoperative epilepsy, and postoperative epileptological outcomes.

Histology	*N*	Preoperative epilepsy			EPS	ILAE class 1 at 6 months*		ILAE class 1 at 24 months^*^	
		No seizures	Seizures, but not DRE	DRE		All	w/epilepsy	All	w/epilepsy
Glioblastom	38	22/38 (57.9%)	16/38 (42.1%)	0	2/38 (14.3%)	28/31 (90.3%)	10/12 (83.3%)	26/28 (92.9%)	8/10 (80.0%)
Gliomas CNS WHO grades 2–3	36	7/36 (19.4%)	28/36 (77.8%)	1/36 (2.8%)	6/36 (16.7%)	30/33 (90.9%)	23/26 (88.5%)	31/32 (97.0%)	24/26 (92.3%)
Other gliomas	7	1/7 (14.3%)	0	6/7 (85.7%)	1/7 (14.3%)	6/7 (85.7%)	5/6 (83.3%)	5/7 (71.4%)	4/6 (66.7%)
Glio-neuronal tumors	73	4/73 (5.5%)	2/73 (2.7%)	67/73 (91.8%)	5/73% (6.8%)	61/73 (83.6%)	57/69 (82.6%)	58/73 (79.5%)	51/69 (73.9%)
Developmental lesions	3	0	1/3 (33.3%)	2/3 (66.7%)	0/3 (0%)	2/3 (66.6%)	2/3 (66.6%)	1/3 (33.3%)	1/3 (33.3%)
All	157	34/157 (21.7%)	47/157 (29.9%)	76/157 (48.4%)	14/157 (8.9%)	127/147 (86.4%)	97/116 (83.6%)	122/144 (84.7%)	88/114 (77.2%)

*Excluding EPS. DRE, drug-resistant epilepsy; ILAE, International League Against Epilepsy.

Overall, 14/157 (8.9%) suffered an EPS (early postoperative seizure, Table 5) (14). One hundred and twenty-seven of 147 assessable patients (86.4%) reported no seizures or auras at 6 months (excluding EPS). A total of 122 (84.7%) of 144 assessable cases were seizure- and aura-free at 24 months. ILAE class 1 outcomes were seen in 90.0 and 92.1% of cases with preoperative epilepsy but not DRE at 6 and 24 months, respectively. The corresponding figures for all cases with DRE were 80.3 and 69.7% (Engel class 1: 84.2 and 77.6%). 2/7 (42.9%) of cases with DRE and residual tumor reported recurrent seizures at 24 months (*cf.* 16/69 [23.2%] in DRE patients with no tumor remnants). Epilepsy surgery outcomes varied surprisingly little with tumor histology and the anatomical compartments infiltrated by the tumors (Tables 4, 5).

### 3.6 Survival outcomes

Overall, 29 patients were followed until death (including 24/38 glioblastomas/astrocytomas CNS WHO grade 4). Median follow-up in the remainder was 37.8 (IQR: 24.4–64.5; mean 45.6 ± 29.6) months.

**Figure 5:**
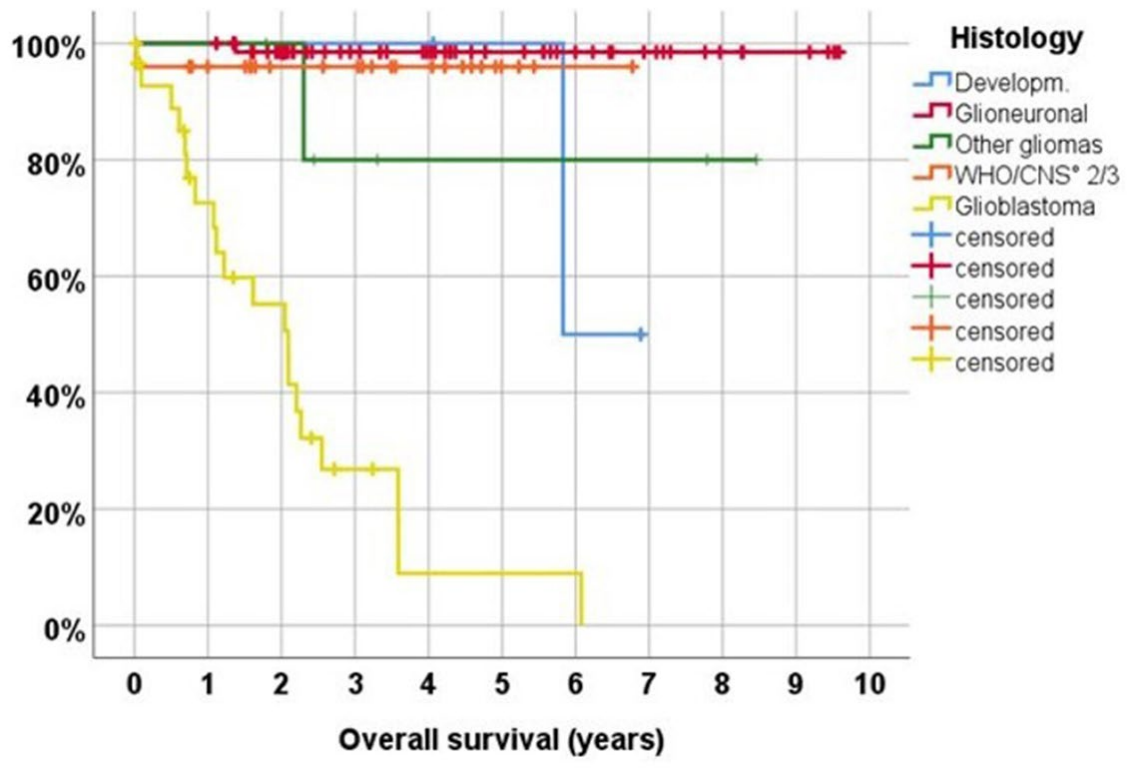
Overall survival following first surgery in 133 cases. Developm., glioneuronal or vascular hamartoma; Glioneuronal, gangliocytoma and ganglioglioma CNS WHO grade 1, atypical and anaplastic ganglioglioma CNS WHO grades 2/3, dysembryoplastic neuroepithelial tumor (DNT); other gliomas, pilocytic astrocytoma CNS WHO grade 1, isomorphic astrocytoma CNS WHO grade 1, angiocentric glioma CNS WHO grade 1; CNS WHO ° 2/3, anaplastic astrocytoma CNS WHO grade 3, diffuse astrocytoma CNS WHO grade 2, (anaplastic) oligodendroglioma CNS WHO grades 2/3; Glioblastoma, IDH wildtype glioblastoma CNS WHO grade 4, IDH mutant astrocytoma CNS WHO grade 4.

Kaplan Meier estimates for 5-year overall survival were 98.5% for non-recurrent glioneuronal tumors, and the 2-year overall survival estimates were 96.0% for 24 primary diffuse CNS WHO grade 2 and 3 gliomas and 55.2% following 30 first surgeries for glioblastomas/astrocytomas CNS WHO grade 4 (Figure 5). Estimated overall survival at 2 and 5 years was 83.8 and 69.6%, respectively, in 28 cases with insular resections for primary tumors (8 glioblastomas, 1 IDH1 mutant astrocytoma CNS WHO grade 4, 3 CNS WHO grade 3 and 8 CNS WHO grade 2 astrocytomas, 6 oligodendrogliomas CNS WHO grades 2/3, 1 ganglioglioma and 1 dysembryoplastic neuroepithelial tumor CNS WHO grade 1).

There was a strong correlation between the extent of resection and survival. Estimated 2-year overall survival in non-recurrent cases was 96.3% following a supramarginal, 78.5% after a complete/gross total resection, and 75.8% in cases with residual tumor (*p* < 0.001). Statistical significance was, however, lost after stratification for tumor histology.

## 4 Discussion

Surgical management of paralimbic brain tumors involving the temporal lobe needs to address both neuro oncological as well as epileptological issues. For the present study, we have reviewed our experience with an anatomy-guided modular concept for the surgical treatment of such lesions that reflects an epilepsy surgery perspective and tumor surgery concepts. We found that our overall approach was generally safe and was associated with good epilepsy control. Resection outcomes were also quite good, which may have had a positive impact on patient survival.

Briefly, tumors were removed by resecting defined tumor-infiltrated anatomical compartments rather than the tumor inside-out. The definition of the compartments reflects the surgical maneuvers necessary for their removal. It combines certain basic operations performed for the treatment of temporal lobe epilepsy, such as temporal pole resection, anterior temporal lobectomy, and removal of the temporo-mesial structures with or without preservation of the body of the hippocampus (7) with the principles of tumor surgery for insular tumors (3–6). Of note, removing certain brain compartments that are infiltrated by a tumor rather than operating just the tumor results in supramarginal resections (10–12). From an epilepsy surgery point of view, supramarginal resections are very similar to extended lesionectomies, which are often performed as surgical treatment for tumor-related epilepsy (7).

Tumor location may play a major role in the pathophysiology of tumor-associated epilepsy (18), and the overall high incidence of seizures in our cohort seems to be in accordance with the assumption that the temporo-insular region is particularly epileptogenic (19). Tumor infiltration of the various anatomical compartments varied with epilepsy history. Of note, presentation with medication-resistant epilepsy correlated with infiltration of the (anterior) temporo-mesial rather than other paralimbic or temporo-lateral regions. To some extent, this might be explained by the fact that patients with glioneuronal tumors presented most often with drug-resistant epilepsy. The tumors invariably involved the anterior temporo-mesial compartment, i.e., the correlations between epilepsy history and involvement of certain parts of the brain may very well reflect the preferential growth patterns of the various tumor types and vice versa.

Patients with glioblastomas were older, presented more often with neurological deficits and less often with seizures, and the growths commonly infiltrated the lateral temporal lobe. Insular and other paralimbic tumor infiltration was most commonly seen in diffuse CNS grade 2–3 gliomas. It is quite possible that the differential growth patterns seen in diffuse gliomas of different CNS WHO grades, at least in part, reflect more selective surgical indications in patients with glioblastomas. Per routine, we have been hesitant to recommend surgical debulking of insular glioblastoma. Therefore, we have performed biopsies rather than resections in cases with temporo-insular glioblastomas when a meaningful tumor resection would have included major insular surgery (3, 6).

Two patients died during their hospital stay, and major neurological deficits at discharge were seen in four cases, i.e., surgery for paralimbic temporo-insular tumors is reasonably safe. Interestingly, all of these patients had insular surgery for diffuse gliomas, and there was an association with surgery for recurrent disease. These findings are not unexpected (3–6) but help with surgical decision-making, e.g., neurological sequelae are not a major concern in most cases with presumed glioneuronal tumors, while insular and repeat tumor surgery carries increased risks.

Epilepsy control rates were quite good following epilepsy surgery for drug-resistant epilepsy. Using the fairly strict ILAE definitions, 53/76 (69.7%) of our patients were completely seizure-free at 2 years. Engel class 1 outcomes (no disabling seizures) were seen in 77.6% at 2 years, which is in line with the current literature, including a recently published multicenter cohort study (20, 21).

Importantly, we observed that there were only 5/63 (7.9%) de-novo or recurring seizures in the first two years following surgery, in the remainder of our cases. This figure compares quite favorably with other series. In a very recent study, Stritzelberger and co-workers described a 36.1% seizure incidence following surgery (19.2% biopsies) in 421 glioblastoma cases (22). Ollila and Roivainen (23) report 123 cases with diffuse gliomas CNS WHO grades 2–4 who had resective or bioptic (15.4%) surgery between 2013 and 2015. 70.7% presented with tumor-associated epilepsy. Overall, only 57.6% of their cases were seizure-free for at least 12 months at some point during follow-up. Carstam (24) and co-workers described 67.5% Engel class 1a (= ILAE class 1) outcomes (completely seizure-free) in a series of 130 lower-grade gliomas, and only 56.8% of cases were reportedly seizure-free (i.e., free of disabling seizures, Engel class 1) for one year or more in the low-grade glioma series by Solomons et al. (25).

Since a large proportion of our patients had more extensive surgery than a simple tumor resection, our data may suggest that removing adjacent (and potentially epileptogenic) brain tissues in tumor cases, i.e., an “extended lesionectomy” or even a temporal lobectomy, will result in better epilepsy outcomes when compared to just resecting the tumor. Indeed, Borger et al. (9) reported a 100% (13/13 cases) vs. 50% (10/20 cases) seizure freedom rate following a temporal lobectomy vs. tumor resection only in patients with temporal lobe glioblastoma.

Survival rates were also quite good in our cohort. While a 98.5% 5-year overall survival rate in cases with glioneuronal tumors is somewhat expected (17, 21, 26), 2-year survival rates of 96.0% in 36 diffuse CNS WHO grade 2/3 astrocytomas and oligodendrogliomas (grade 2: 13, grade 3: 23) and 55.2% in glioblastoma patients are very good. For comparison, the prospective trial that evaluated the use of tumor-treating fields as part of the primary treatment of glioblastomas reported a 43% 2-year survival in the experimental treatment arm (27). Five-year overall survival rates in anaplastic gliomas varied between 40 and 50% in the CATNON, EORTC 26951, and RTOG 9402 phase III trials (28, 29) and between 85 and 90% in some larger CNS WHO grade 2 glioma cohorts reported in the literature (30, 31).

Undoubtedly, our results reflect confounding factors such as age and the presence of epilepsy. Epilepsy has been linked to improved survival in several glioma cohorts (32, 33). Additionally, some data suggest that gliomas involving the insula are generally associated with a better prognosis (5). However, another possible explanation for the encouraging overall survival rates in our study could be the relatively high rate of successful resections (63.6% in all non-recurrent diffuse glioma cases), despite the frequent involvement of the insula, basal ganglia, or diencephalon. The literature reports rates of complete or gross total resections versus partial resections for non-recurrent diffuse gliomas not selected by location ranging from 20 to 60% (24, 27, 30, 31). In our series, resection outcomes were strongly correlated with patient survival, with the best survival outcomes observed following supramarginal resections. However, this latter finding may be influenced by the correlation between tumor histology and preferential growth patterns, which significantly affect resectability. Specifically, the most favorable resection outcomes were seen in surgeries for benign glioneuronal tumors.

Our study has clear limitations. It is based on a retrospective review of a mono-institutional experience, and the cohort is heterogeneous in terms of histology, tumor growth patterns, age, and presentation. Additionally, the availability of epilepsy surgery alongside neuro-oncological services introduces a selection bias.

## 5 Conclusion

Combining epilepsy and tumor surgery techniques for treating a cohort of unselected brain tumors involving the mesial temporal lobe and, to varying degrees, the insula led to more extensive resections, improved seizure outcomes, and potentially enhanced patient survival outcomes. Temporal lobe resections were associated with very low neurological risks. However, additional removal of the insular tumor, often necessary for optimal resection—and thus improved survival and seizure outcomes—carries significant neurological risks.

## Data Availability

The raw data supporting the conclusions of this article will be made available by the authors, without undue reservation.
